# Sex differences in prevalence and clinical correlates for psychotic symptoms in first-episode drug-naïve patients with bipolar disorder: a cross-sectional study

**DOI:** 10.1186/s12991-026-00654-5

**Published:** 2026-04-07

**Authors:** Yehong Chen, Yujia Zhai, Hao Chen, Xuebing Liu

**Affiliations:** 1 Affiliated Wuhan Mental Health Center, Tongji Medical College of Huazhong University of Science and Technology, No. 89, Gongnongbing Road, Jiang′an District, Wuhan, 430012 Hubei province China; 2 Department of Psychiatry, Wuhan Mental Health Center, Wuhan, 430012 Hubei province China; 3 Department of Psychiatry, Wuhan Hospital for Psychotherapy, Wuhan, 430012 Hubei province China; 4 Department of Psychiatry, National Clinical Research Center for Mental Disorders, the Second Xiangya Hospital of Central South University, Changsha, 410011 Hunan China

**Keywords:** Sex differences, Bipolar disorder, Psychotic symptoms, High-density lipoprotein cholesterol, Thyroid-stimulating hormone, Metabolic syndrome

## Abstract

**Background:**

Psychotic symptoms, which affect a significant proportion of patients with bipolar disorder (BD), are associated with adverse prognostic outcomes. Currently, sex-specific patterns of psychotic symptoms remain inadequately characterized in BD. This study seeks to investigate sex-based differences in psychotic symptoms among first-episode drug-naïve (FEDN) BD patients among Han Chinese population.

**Methods:**

A total of 577 FEDN BD patients, comprising 271 males and 306 females, were recruited in this study. Demographic characteristics were collected using a specialized questionnaire. The positive subscale of the Positive and Negative Syndrome Scale (PANSS), Hamilton Depression Scale, Young Mania Rating Scale, and Clinical Global Impression Scale-Severity of Illness were used to evaluate clinical symptoms. Moreover, fasting blood glucose, prolactin, lipids, and thyroid hormone levels, alongside metabolic syndrome (MetS) were assessed.

**Results:**

The prevalence of psychotic symptoms showed no significant sex differences in the FEDN BD cohort (*p* = 0.968). Nevertheless, multiple linear regression revealed that MetS score (*p* = 0.001) was significantly associated with the PANSS Positive subscale score (PANSS-P) among males, whereas thyroid-stimulating hormone (TSH) levels (*p* < 0.001) and high-density lipoprotein cholesterol (HDL-C) levels (*p* < 0.001) were significantly associated with PANSS-P among females. Binary logistic regression revealed that MetS score (*p* = 0.002) was a significant correlate of the prevalence of psychotic symptoms among males only, whereas TSH levels (*p* < 0.001) significantly correlated with the prevalence of psychotic symptoms among females only.

**Conclusions:**

Among Han Chinese FEDN BD patients, our study revealed the following: (1) No significant sex difference was observed in the prevalence of psychotic symptoms; (2) HDL-C functions as a meaningful protective factor against the prevalence of psychotic symptoms in both males and females, while its association with symptom severity is observed exclusively in females; (3) Sex-specific indicators correlate with psychotic symptoms, specifically MetS dominates in males, while TSH in females. Our results call for sex-informed clinical protocols that integrate metabolic-endocrine monitoring and targeted interventions to reduce psychosis risk in BD.

## Introduction

Bipolar disorder (BD) is a chronic, debilitating psychiatric disorder characterized by recurrent episodes of depression and mania or hypomania [1], and is recognized as one of the leading causes of disability worldwide [2]. According to the Global Burden of Disease Study 2019, approximately 0.53% of the global population (40 million individuals) were diagnosed with BD, highlighting a rising trend in prevalence and global burden [3]. Notably, individuals with BD exhibit a two- to three-fold elevated risk of premature mortality compared to the general population [4, 5], with a concomitant life expectancy reduction of 10–20 years [6, 7]. Reduction in life expectancy is primarily due to suicide, which is strongly associated with psychotic symptoms [8].

Notably, approximately 50% to 70% of patients with BD develop psychotic symptoms—which typically manifest as either delusions or hallucinations—during their lifetime [9, 10]. The presence of psychotic symptoms has been associated with poor long-term outcomes in patients with BD [11, 12], often characterized by poor functional outcomes, substantial cognitive impairments, and poor general health comorbid with more somatic illnesses [12–14]. Additionally, psychotic symptoms have been identified as a predictor of prolonged inpatient hospitalization [15]. Notably, psychotic symptoms in BD exhibit no qualitative distinction from those observed in schizophrenia [10, 16, 17], and their high prevalence especially during manic episodes complicates the differential diagnosis from primary psychotic disorders [18].

Although sex-related differences in the epidemiology, clinical phenomenology, illness course, and other clinical characteristics of BD have been established [19–21], sex-specific patterns of psychotic symptoms remain inadequately characterized. Existing studies report conflicting results: while some studies indicate no sex differences in the prevalence of psychotic symptoms [22], others show that female patients are more predisposed to psychotic symptoms [23, 24]. Furthermore, female patients may experience greater severity of psychotic symptoms [25, 26]. Research has also revealed sex differences in accompanying episode and symptom types [10, 22, 27]. Emerging evidence suggests that certain endocrine and metabolic indicators—including thyroid axis hormones [28, 29], lipids [30, 31], and metabolic syndrome (MetS) [32]—which exhibit sex-specific profiles, potentially contributed to the risk of psychotic symptoms in BD. While there are indications that these markers demonstrate sex-dependent associations with psychotic symptoms in BD, the underlying mechanisms remain to be elucidated in detail. Particularly, the sex-based baseline differences and the potential impact of drug treatment should be taken into account [33].

To date, sex differences in prevalence and clinical correlates of psychotic symptoms remain unexplored among the Han Chinese population with BD. This study tests the hypothesis that psychotic symptoms in BD differ significantly between sexes. By analyzing a cohort of first-episode drug-naïve (FEDN) patients with BD, we investigate sex-based differences in psychotic symptoms and explore factors that may contribute to any identifiable phenomenon. Elucidating these underlying mechanisms is critical to developing sex-specific clinical assessment and intervention. Ultimately, our findings aim to advance research on concurrent correlates and precision medicine approaches for BD by identifying modifiable, sex-informed therapeutic targets.

## Methodology

This study was conducted at Wuhan Mental Health Center and Nanyang Mental Health Center from 2016 to 2024. Consecutive sampling was used to recruit participants from the outpatient and inpatient units. The inclusion criteria were as follows: (1) Patients meeting BD diagnostic criteria as outlined by the International Classification of Diseases, 10th edition (ICD-10) and confirmed by two qualified psychiatrists using the Mini-International Neuropsychiatric Interview (MINI) standard [34]; (2) not receiving any psychotropic medication, including antipsychotics, mood stabilizers, or antidepressants; (3) Chinese Han individuals aged between 18 and 60 years; (4) the total score in the 17-item Hamilton Depression Scale (HAMD) ≥ 7 points and/or the Young Mania Rating Scale (YMRS) score ≥ 6 points. The exclusion criteria were as follows: (1) presence of any other psychiatric disorders, including schizophrenia, major depressive disorder, substance use disorder, and intellectual disability; (2) presence of severe physical diseases, such as organic brain diseases, chronic infections, and autoimmune disorders; (3) female participants who were pregnant, breastfeeding, or had pregnancy intentions.

The study was approved by the Ethics Committee of Wuhan Mental Health Center (Approval No: KY2022090306) and Nanyang Mental Health Center (Approval No: 2016-BHD003). Prior to enrollment, informed consent was obtained from all participants or their legal guardian (in cases of impaired capacity due to acute psychiatric symptoms). Study procedures were explained in plain language, and sufficient time was provided for consideration.

### Assessment

All participants were evaluated using the following scales and instruments: (1) Positive subscale of the Positive and Negative Syndrome Scale (PANSS) [35], comprising items P1-P7, was used to assess psychotic symptoms. The PANSS Positive subscale score (PANSS-P, range 7–49) is used to measure the severity of psychotic symptoms. For exploratory analysis, a total score ≥ 15 was used to categorize patients as having psychotic symptoms [36]; (2) the HAMD was used to assess the levels of depression [37]; (3) the YMRS was used to evaluate the levels of manic symptoms [38]; (4) the Clinical Global Impression Scale-Severity of Illness (CGI-SI) was used to assess overall illness severity [39]. All assessments were conducted by the same psychiatrists, who had completed standardized training. After repeated assessments, all the correlation coefficients for the scales were found to exceed 0.8.

### Demographic characteristics

Specially designed questionnaires were used to collect demographic characteristics of all patients, including sex, age, education level, marital status, age of disease onset, duration of illness, and family history of mental disorders. Additionally, the history of substance abuse was directly reported by either the patient or their family members.

### Blood samples and measurements

Blood samples were collected from each participant between 06:00 am and 08:00 am following overnight fasting. Lipids and endocrine markers were quantified, including glucose, total cholesterol (TC), triglycerides (TG), high-density lipoprotein cholesterol (HDL-C), low-density lipoprotein cholesterol (LDL-C), thyroid-stimulating hormone (TSH), free triiodothyronine (FT_3_), free thyroxine (FT_4_), and prolactin (PRL).

Anthropometric measurements were recorded using calibrated instruments following standardized protocols: (1) height was measured to nearest 0.1 cm; (2) weight was measured to nearest 0.1 kg; (3) waist circumference was measured at the midway between iliac crest and lowest rib margin at end-expiration in the horizontal plane, with an accuracy of 0.1 cm; (4) blood pressure (BP), including resting systolic BP and diastolic BP were measured in mmHg; (5) body mass index (BMI) was determined as weight/height² (kg/m^2^).

### Diagnostic criteria of MetS

The diagnostic criteria for MetS based on the Chinese Diabetes Society [40] were as follows: (1) abdominal/central obesity was defined as waist circumference ≥ 90 cm in men and ≥ 85 cm in women; (2) dysglycemia was defined as fasting blood glucose ≥ 6.1 mmol/L or 2-hour postprandial glucose ≥ 7.8 mmol/L, and/or individuals diagnosed and treated for diabetes; (3) hypertension was defined as BP ≥ 130/85 mmHg and/or individuals diagnosed and treated for hypertension; (4) individuals with fasting TG ≥ 1.70 mmol/L; (5) individuals with fasting HDL-C < 1.04 mmol/L. The diagnosis of MetS required meeting three or more of these criteria. To quantify the metabolic burden on a continuous scale, we calculated a MetS score based on a validated calculation method developed for Chinese adults [41].

### Data analysis

Data normality was assessed using the Shapiro-Wilk test. Continuous variables were reported as mean ± standard deviation (SD), while categorical variables were reported as frequencies (%). The chi-square test and independent samples t-tests were used for group comparison for categorical and continuous variables, respectively. A two-way ANOVA was used to assess the interaction between psychotic symptoms at two levels (with and without psychotic symptoms) and sex at two levels (male and female). Spearman’s correlation and multiple linear regression analyses with collinearity analysis were used to evaluate the factors associated with PANSS-P for male and female patients. Binary logistic regression was used to investigate the correlates associated with the prevalence of psychotic symptoms in male and female patients. The Bonferroni correction was applied to adjust for multiple comparisons. All analyses were two-tailed with *p*-value < 0.05. Statistical analysis was performed using SPSS version 27.0.

## Results

### Sex differences in the demographic and clinical characteristics in BD

Demographic and clinical characteristics of patients with BD, stratified by sex, are summarized in Table 1. Comparison between the two groups revealed that female patients exhibited a significantly higher level of marriage rates (*χ*^*2*^ = 8.989, *p* = 0.003), PRL (*t* = −4.964, *p* < 0.001), HDL-C (*t* = −5.440, *p* < 0.001), and MetS score (*t* = −6.999, *p* < 0.001). Conversely, male patients exhibited significantly higher likelihood of later age of onset (*t* = 2.070, *p* = 0.039), higher FT_3_ levels (*t* = 1.992, *p* = 0.047), and higher BMI (*t* = 3.790, *p* < 0.001) compared to female patients. Notably, no significant sex-related difference was observed in the prevalence of psychotic symptoms among BD patients, with 13.3% and 13.4% of male and female patients, respectively, exhibiting psychotic symptoms (*χ*^*2*^ = 0.002, *p* = 0.968). After Bonferroni correction, significant sex-related differences persisted in marriage status, PRL, HDL-C, BMI, and MetS score. However, no sex-related differences were observed in the remaining variables (all *p* > 0.05).

**Table 1:** Sex differences in the demographic and clinical characteristics of patients with BD

Variables	Total patients (*N* = 577)	Male (*N* = 271)	Female (*N* = 306)	*χ*^*2*^/*t*	*p*-value
**Education level**				2.145	0.543
Junior high school	165 (28.6%)	85 (31.4%)	80 (26.1%)		
High school	180 (31.2%)	82 (30.3%)	98 (32.0%)		
Bachelor	212 (36.7%)	96 (35.4%)	116 (37.9%)		
Master	20 (3.5%)	8 (3.0%)	12 (3.9%)		
**Marital status**				8.989	**0.003**
Single	399 (69.2%)	204 (75.3%)	195 (63.7%)		
Married	178 (30.8%)	67 (24.7%)	111 (36.3%)		
**Family history of mental disorders**				0.061	0.805
No	418 (72.4%)	195 (72%)	223 (72.9%)		
Yes	159 (27.6%)	76 (28%)	83 (27.1%)		
**MetS**				1.793	0.181
No	479 (83.0%)	231 (85.2%)	248 (81.0%)		
Yes	98 (17.0%)	40 (14.8%)	58 (19.0%)		
**Psychotic symptoms**				0.002	0.968
No	500 (86.7%)	235 (86.7%)	265 (86.6%)		
Yes	77 (13.3%)	36 (13.3%)	41 (13.4%)		
**Age**,** years**	26.70 ± 11.55	26.86 ± 11.66	26.56 ± 11.46	0.312	0.755
**Age of onset**,** years**	21.15 ± 9.18	21.99 ± 9.58	20.41 ± 8.75	2.070	**0.039**
**Illness duration**,** years**	6.48 ± 6.57	5.99 ± 5.99	6.92 ± 7.02	−1.708	0.088
**HAMD**	19.18 ± 2.66	19.21 ± 2.75	19.15 ± 2.58	0.256	0.798
**YMRS**	17.56 ± 1.49	17.58 ± 1.52	17.55 ± 1.46	0.218	0.828
**PANSS-P**	9.71 ± 4.13	9.57 ± 4.09	9.83 ± 4.17	−0.758	0.449
**CGI-SI**	5.52 ± 0.62	5.52 ± 0.62	5.52 ± 0.63	0.005	0.996
**TSH**,** uIU/mL**	2.81 ± 3.91	2.63 ± 4.41	2.97 ± 3.41	−1.024	0.306
**FT**_**3**_, **pmol/L**	3.10 ± 1.10	3.19 ± 0.79	3.01 ± 1.31	1.992	**0.047**
**FT**_**4**_, **pmol/L**	1.04 ± 0.35	1.05 ± 0.38	1.03 ± 0.32	0.649	0.516
**PRL**,** ng/ml**	21.33 ± 27.38	15.65 ± 16.03	26.36 ± 33.68	−4.964	**< 0.001**
**TG**,** mmol/L**	1.33 ± 0.90	1.41 ± 0.97	1.27 ± 0.83	1.876	0.061
**TC**,** mmol/L**	4.27 ± 1.79	4.17 ± 0.86	4.36 ± 2.32	−1.301	0.194
**HDL-C**,** mmol/L**	1.23 ± 0.31	1.16 ± 0.29	1.30 ± 0.31	−5.440	**< 0.001**
**LDL-C**,** mmol/L**	2.20 ± 0.71	2.20 ± 0.73	2.19 ± 0.70	0.220	0.826
**BMI**,** kg/m²**	22.90 ± 4.23	23.60 ± 4.28	22.28 ± 4.09	3.790	**< 0.001**
**MetS score**	0.03 ± 0.65	−0.16 ± 0.59	0.20 ± 0.65	−6.999	**< 0.001**

MetS = metabolic syndrome; HAMD = Hamilton Depression Rating Scale; YMRS = Young Mania Rating Scale; PANSS-P = the PANSS Positive subscale; CGI-SI = Clinical Global Impression Scale-Severity of Illness; TSH = thyroid stimulating hormone; FT_3_ = free triiodothyronine; FT_4_ = free thyroxine; PRL = Prolactin; TG = triglycerides; TC = total cholesterol; HDL-C = high density lipoprotein cholesterol; LDL-C = low density lipoprotein cholesterol; BMI = Body Mass Index. The significance of bold emphases in the table is *p* < 0.05

### Differential main effects of sex and psychotic symptoms on the demographics and clinical characteristics without significant interaction in BD

Table 2 presents two-way ANOVA results examining the interaction between psychotic symptoms and sex. The results revealed significant effects of psychotic symptoms at two levels (present or absent) on HAMD score (*F* = 54.903, *p* < 0.001), YMRS score (*F* = 16.340, *p* < 0.001), CGI-SI score (*F* = 35.421, *p* < 0.001), TSH levels (*F* = 5.609, *p* = 0.018), TG levels (*F* = 26.987, *p* < 0.001), HDL-C levels (*F* = 73.958, *p* < 0.001), BMI (*F* = 8.099, *p* = 0.005), and MetS score (*F* = 51.549, *p* < 0.001). Significant effects of sex at two levels (male or female) were observed for PRL levels (*F* = 10.304, *p* = 0.001), HDL-C levels (*F* = 13.843, *p* < 0.001), BMI (*F* = 4.866, *p* = 0.028), and MetS score (*F* = 14.585, *p* < 0.001). However, psychotic symptoms and sex did not exert significant effect on the remaining variables (all *p* > 0.05). Moreover, no significant effect was observed in the interaction between psychotic symptoms and sex (all *p* > 0.05).

**Table 2:** Demographic and clinical characteristics of BD patients with and without psychotic symptoms, grouped by sex

Variables	Without PS (*N* = 500)		With PS (*N* = 77)		Sex *F* (*p*-value)	Subgroup *F *(*p*-value)	Sex × subgroup *F* (*p*-value)
	Male (*N* = 235)	Female (*N* = 265)	Male (*N* = 36)	Female (*N* = 41)			
**Age**,** years**	26.69 ± 11.65	26.75 ± 11.45	27.97 ± 11.83	25.29 ± 11.56	0.767 (0.381)	0.001 (0.974)	1.018 (0.313)
**Age of onset**,** years**	22.02 ± 9.73	20.42 ± 8.89	21.75 ± 8.63	20.29 ± 7.92	1.754 (0.186)	0.025 (0.876)	0.001 (0.973)
**Illness duration**,** years**	5.91 ± 6.06	7.02 ± 7.27	6.56 ± 5.55	6.24 ± 5.11	0.272 (0.603)	0.003 (0.957)	0.824 (0.365)
**HAMD**	18.89 ± 2.55	18.86 ± 2.51	21.33 ± 3.09	21.05 ± 2.19	0.241 (0.624)	**54.903 (< 0.001)**	0.175 (0.676)
**YMRS**	17.69 ± 1.45	17.64 ± 1.40	16.86 ± 1.76	17.00 ± 1.75	0.058 (0.810)	**16.340 (< 0.001)**	0.279 (0.597)
**CGI-SI**	5.47 ± 0.57	5.45 ± 0.56	5.83 ± 0.81	5.98 ± 0.82	0.650 (0.420)	**35.421 (< 0.001)**	1.217 (0.270)
**TSH**,** uIU/mL**	2.56 ± 4.72	2.74 ± 3.59	3.14 ± 0.93	4.42 ± 1.20	2.304 (0.130)	**5.609 (0.018)**	1.325 (0.250)
**FT**_**3**_, **pmol/L**	3.19 ± 0.81	2.99 ± 1.40	3.25 ± 0.68	3.15 ± 0.41	1.157 (0.283)	0.623 (0.430)	0.117 (0.732)
**FT**_**4**_, **pmol/L**	1.03 ± 0.31	1.03 ± 0.33	1.17 ± 0.68	1.06 ± 0.25	1.842 (0.175)	3.443 (0.064)	1.469 (0.226)
**PRL**,** ng/ml**	16.17 ± 16.85	27.00 ± 34.72	12.24 ± 8.31	22.22 ± 25.98	**10.304 (0.001)**	1.840 (0.176)	0.037 (0.847)
**TG**,** mmol/L**	1.33 ± 0.94	1.20 ± 0.85	1.90 ± 0.98	1.72 ± 0.58	2.101 (0.148)	**26.987 (< 0.001)**	0.052 (0.820)
**TC**,** mmol/L**	4.19 ± 0.86	4.38 ± 2.47	4.05 ± 0.80	4.24 ± 0.87	0.760 (0.384)	0.418 (0.518)	0 (0.986)
**HDL-C**,** mmol/L**	1.20 ± 0.27	1.34 ± 0.30	0.91 ± 0.26	1.03 ± 0.27	**13.843 (< 0.001)**	**73.958 (< 0.001)**	0.167 (0.683)
**LDL-C**,** mmol/L**	2.20 ± 0.73	2.16 ± 0.68	2.23 ± 0.70	2.36 ± 0.82	0.325 (0.569)	1.694 (0.194)	1.010 (0.315)
**BMI**,** kg/m²**	23.45 ± 4.22	22.06 ± 3.88	24.55 ± 4.61	23.71 ± 5.06	**4.866 (0.028)**	**8.099 (0.005)**	0.293 (0.588)
**MetS score**	−0.25 ± 0.51	0.15 ± 0.65	0.38 ± 0.76	0.55 ± 0.49	**14.585 (< 0.001)**	**51.549 (< 0.001)**	2.534 (0.112)

MetS = metabolic syndrome; HAMD = Hamilton Depression Rating Scale; YMRS = Young Mania Rating Scale; CGI-SI = Clinical Global Impression Scale-Severity of Illness; TSH = thyroid stimulating hormone; FT_3_ = free triiodothyronine; FT_4_ = free thyroxine; PRL = Prolactin; TG = triglycerides; TC = total cholesterol; HDL-C = high density lipoprotein cholesterol; LDL-C = low density lipoprotein cholesterol; BMI = Body Mass Index. The significance of bold emphases in the table is *p* < 0.05

### Sex Differences in related factors of psychotic symptoms severity in BD

In the subsequent analysis, we stratified the related factors by sex. Table 3 presents the results of correlation analyses for PANSS-P involving male and female patients separately. Among female patients, PANSS-P exhibited significant correlations with HAMD score, YMRS score, CGI-SI score, TSH levels, FT_3_ levels, FT_4_ levels, TG levels, HDL-C levels, and MetS score (all *p* < 0.05). Correspondingly, PANSS-P among males showed significant associations with all the above-mentioned variables except for FT_4_ levels (all *p* < 0.05).

Subsequently, we performed multiple regression analysis to analyze the variables that were independently significantly correlated with PANSS-P among males and females. As shown in Table 4, multiple regression analysis showed that among males, HAMD score (*B* = 0.706, *t* = 3.659, *p* < 0.001), YMRS score (*B* = 0.817, *t* = 2.395, *p* = 0.017), CGI-SI score (*B* = 1.052, *t* = 2.883, *p* = 0.004), and MetS score (*B* = 1.962, *t* = 3.260, *p* = 0.001) were independently associated with PANSS-P. Additionally, among females, HAMD score (*B* = 1.585, *t* = 6.798, *p* < 0.001), YMRS score (*B* = 2.158, *t* = 5.303, *p* < 0.001), CGI-SI score (*B* = 1.268, *t* = 3.998, *p* < 0.001), TSH levels (*B* = 0.208, *t* = 3.531, *p* < 0.001), and HDL-C levels (*B* = −3.357, *t* = 4.188, *p* < 0.001) were independently associated with PANSS-P. Variance Inflation Factors (*VIF*s) for all independent variables in the regression model showed values < 10, indicating no significant multicollinearity. After adjusting for potential confounders, these statistically significant factors remained related factors of PANSS-P (all *p* < 0.05).

**Table 3:** Correlation between PANSS-P and demographic and clinical characteristics in male and female patients with BD

Variables	Male (*N* = 271)	Female (*N* = 306)
	*r* (*p*-value)	*r* (*p*-value)
**Age**,** years**	−0.026 (0.674)	−0.067 (0.240)
**Age of onset**,** years**	0.027 (0.653)	0.013 (0.821)
**Illness duration**,** years**	−0.070 (0.248)	−0.106 (0.064)
**HAMD**	**0.205 (< 0.001)**	**0.344 (< 0.001)**
**YMRS**	**−0.132 (0.030)**	**−0.249 (< 0.001)**
**CGI-SI**	**0.175 (0.004)**	**0.181 (0.002)**
**TSH**,** uIU/mL**	**0.179 (0.003)**	**0.253 (< 0.001)**
**FT**_**3**_, **pmol/L**	**0.227 (< 0.001)**	**0.242 (< 0.001)**
**FT**_**4**_, **pmol/L**	0.029 (0.639)	**0.127 (0.026)**
**PRL**,** ng/ml**	−0.011 (0.860)	−0.073 (0.203)
**TG**,** mmol/L**	**0.179 (0.003)**	**0.188 (< 0.001)**
**TC**,** mmol/L**	−0.087 (0.155)	−0.071 (0.214)
**HDL-C**,** mmol/L**	**−0.262 (< 0.001)**	**−0.239 (< 0.001)**
**LDL-C**,** mmol/L**	0.043 (0.483)	0.014 (0.815)
**BMI**,** kg/m²**	0.026 (0.667)	0.082 (0.151)
**MetS score**	**0.289 (< 0.001)**	**0.196 (< 0.001)**

MetS = metabolic syndrome; HAMD = Hamilton Depression Rating Scale; YMRS = Young Mania Rating Scale; CGI-SI = Clinical Global Impression Scale-Severity of Illness; TSH = thyroid stimulating hormone; FT_3_ = free triiodothyronine; FT_4_ = free thyroxine; PRL = Prolactin; TG = triglycerides; TC = total cholesterol; HDL-C = high density lipoprotein cholesterol; LDL-C = low density lipoprotein cholesterol; BMI = Body Mass Index. The significance of bold emphases in the table is *p* < 0.05

**Table 4:** Multiple regression analyses of influence factors of PANSS-P in male and female patients with BD

Variables	Male (*N* = 271)			Female (*N* = 306)		
	*B*	*t* (*p*-value)	VIF	*B*	*t* (*p*-value)	VIF
**HAMD**	0.706	**3.659 (< 0.001)**	5.921	1.585	**6.798 (< 0.001)**	9.592
**YMRS**	0.817	**2.395 (0.017)**	5.644	2.158	**5.303 (< 0.001)**	9.439
**CGI-SI**	1.052	**2.883 (0.004)**	1.074	1.268	**3.998 (< 0.001)**	1.055
**TSH**,** uIU/mL**	0.041	0.817 (0.415)	1.014	0.208	**3.531 (< 0.001)**	1.071
**FT**_**3**_, **pmol/L**	0.341	1.223 (0.223)	1.015	0.137	0.898 (0.370)	1.053
**FT**_**4**_, **pmol/L**	-	-	-	0.844	1.314 (0.190)	1.151
**TG**,** mmol/L**	−0.352	−1.177 (0.240)	1.759	0.628	1.383 (0.168)	3.807
**HDL-C**,** mmol/L**	−1.631	−1.627 (0.105)	1.767	−3.357	**−4.188 (< 0.001)**	1.689
**MetS score**	1.962	**3.260 (0.001)**	2.639	−0.433	−0.662 (0.508)	4.764

MetS = metabolic syndrome; HAMD = Hamilton Depression Rating Scale; YMRS = Young Mania Rating Scale; CGI-SI = Clinical Global Impression Scale-Severity of Illness; TSH = thyroid stimulating hormone; FT_3_ = free triiodothyronine; FT_4_ = free thyroxine; TG = triglycerides; HDL-C = high density lipoprotein cholesterol. The significance of bold emphases in the table is *p* < 0.05

### Sex differences in related factors of the prevalence of psychotic symptoms in BD

We investigated sex-specific related factors for the prevalence of psychotic symptoms in patient with BD. Variables exhibiting showing significant associations in the univariate analysis were incorporated into the binary logistic regression analysis models (Backward: Wald) to identify independent related factors for psychotic symptoms among males and females, separately. As shown in Table 5, the related factors for psychotic symptoms among males included: HAMD score (odds ratio (*OR)* = 2.133, 95% confidence interval (CI): 1.331–3.417, *p* = 0.002), YMRS score(*OR* = 2.879, 95% CI: 1.245–6.655, *p* = 0.013), CGI-SI score(*OR* = 2.078, 95% CI: 1.088–3.970, *p* = 0.027), and MetS score (*OR* = 3.092, 95% CI: 1.493–6.402, *p* = 0.002), while lower HDL-C levels (*OR* = 0.039, 95% CI: 0.005–0.326, *p* = 0.003) acted as a protective factor. Correspondingly, the related factors for psychotic symptoms among females included: HAMD score (*OR* = 3.600, 95% CI: 2.032–6.381, *p* = 0.004), YMRS score (*OR* = 6.394, 95% CI: 2.331–17.540, *p* < 0.001), CGI-SI score (*OR* = 2.718, 95% CI: 1.452–5.087, *p* = 0.027), and TSH levels (*OR* = 1.117, 95% CI: 1.033–1.207, *p* < 0.001), whereas lower HDL-C levels (*OR* = 0.034, 95% CI: 0.005–0.244, *p* < 0.001) acted as a protective factor. The related factors for having psychotic symptoms differed significantly by sex: in males, a one-point increase in the MetS score tripled the odds of psychotic symptoms (*OR* = 3.092), whereas in females, a 1 uIU/mL increase in TSH was associated with an 11.7% increase in the odds of psychotic symptoms (*OR* = 1.117). After controlling for potential confounders, these statistically significant factors remained related factors for the prevalence of psychotic symptoms (all *p* < 0.05).

**Table 5:** Binary logistic regression analyses of determinants of the prevalence of psychotic symptoms in male and female patients with BD

Variables	*B*	S.E	Wald	*p*-value	OR	95%CI
**Male (N = 271)**						
** Constant**	−6.315	2.161	8.541	**0.003**	0.000	
** HAMD**	0.757	0.240	9.923	**0.002**	2.133	1.331–3.417
** YMRS**	1.057	0.428	6.116	**0.013**	2.879	1.245–6.655
** CGI-SI**	0.731	0.330	4.905	**0.027**	2.078	1.088–3.970
** TSH**,** uIU/mL**	0.055	0.035	2.452	0.117	1.057	0.986–1.132
** HDL-C**,** mmol/L**	−3.253	1.088	8.931	**0.003**	0.039	0.005–0.326
** MetS score**	1.129	0.371	9.242	**0.002**	3.092	1.493–6.402
**Female (N = 306)**						
** Constant**	−6.564	1.600	16.836	**< 0.001**	0.000	
** HAMD**	1.281	0.292	19.251	**< 0.001**	3.600	2.032–6.381
** YMRS**	1.855	0.515	12.986	**< 0.001**	6.394	2.331–17.540
** CGI-SI**	1.000	0.320	9.777	**0.002**	2.718	1.452–5.087
** TSH**,** uIU/mL**	0.111	0.040	7.730	**0.005**	1.117	1.033–1.207
** HDL-C**,** mmol/L**	−3.377	1.002	11.348	**< 0.001**	0.034	0.005–0.244
** MetS score**	0.222	0.397	0.313	0.576	1.249	0.573–2.719

HAMD = Hamilton Depression Rating Scale; YMRS = Young Mania Rating Scale; CGI-SI = Clinical Global Impression Scale-Severity of Illness; TSH = thyroid stimulating hormone; HDL-C = high density lipoprotein cholesterol; MetS = metabolic syndrome. The significance of bold emphases in the table is *p* < 0.05

## Discussion

Findings from this study hold significant clinical and research implications. Notably, this study revealed the following findings for patients with BD: (1) Female patients exhibit significantly higher marriage rates, PRL levels, HDL-C levels, and MetS scores, while males show elevated BMI; (2) No significant sex difference was observed in **t**he prevalence of psychotic symptoms; (3) HAMD score, YMRS score, CGI-SI score and HDL-C levels are significantly associated with the prevalence of psychotic symptoms among males and females, whereas MetS score and TSH levels correlate with prevalence of psychotic symptoms among males and females only, respectively; (4) HAMD, YMRS, and CGI-SI scores are significantly associated with the serverity of psychotic symptoms in both sexes, while MetS score correlate the serverity of psychotic symptoms only in males, whereas TSH levels and HDL-C levels are associated with the serverity of psychotic symptoms among females only.

Higher marriage rates were observed among female patients with BD, potentially reflecting better psychosocial functioning. Most studies have reported a relationship between sex-specific patterns and psychosocial adjustment in BD [42, 43], with women showing better social and occupational functioning compared to men [44, 45]. Notably, the better psychosocial outcomes for women with BD may be related to sexual dimorphism of brain structures [44, 46], affective symptoms, and other clinical factors [43, 45], However, Maria et al. [47] and Solé et al. [48] found no significant differences in poor psychosocial functioning between sexes. However, this difference may be moderated by cultural factors.

Furthermore, our study showed that there were sex differences in endocrine and metabolic indicators in patients with BD. Notably, PRL levels, HDL-C levels, and MetS scores were significantly higher among females, whereas males exhibited higher BMI. This aligns with the established sexual dimorphism—PRL, a classic estradiol-upregulated pituitary hormone [49], is inherently elevated in post-pubertal females [50]. Importantly, PRL was associated with a higher risk of experiencing mood episodes [51], as well as suicidal ideation and behavior in patients with BD [52, 53], a phenomenon possibly mediated via the dysregulation of the hypothalamus-pituitary system [54]. The sex-based metabolic disparities—involving HDL-C, MetS, and BMI—may be related to sex-specific endocrine-metabolic interactions and behavioral habits. Research reports that female sex hormones positively correlate with HDL-C and negatively correlate with BMI, and present as a significant related factor of MetS [55, 56]. Additionally, elevated health-risk behaviors, including excessive food intake, alcohol consumption, and smoking, may contribute to the higher BMI in men with BD [57]. As identical patterns occur in healthy populations, these observed differences may reflect biological baseline dimorphism rather than BD-specific pathology. Notably, future research needs to incorporate healthy controls to analyze disease-related variations.

One of our primary findings was that the prevalence of psychotic symptoms did not differ significantly by sex in this sample, which represents the first known report among Han Chinese population. This result aligns with findings from some previous studies [22] involving other countries, but contrasts other [23, 24].

Another major finding of this study was that lower HDL-C levels was a significant risk factor for the prevalence of psychotic symptoms in both sexes in patients with BD. Additionally, it was correlated with severity of psychotic symptoms in females only. Research indicates that the HDL-C levels in BD are lower compared to those in healthy control group [30, 58], and the prevalence of psychotic symptoms is potentially attributable to this reduction [59, 60]. Another study reported a significant negative correlation between HDL-C levels and the severity of psychotic symptoms [33, 64]. This correlation may be related to the neuroprotective benefits of HDL-C: First, HDL-C, serving as a proxy for brain cholesterol supply [61],contributes to oligodendrocyte function and myelin synthesis [62–64], thereby influencing neural activity and behavior modulation [65]. Second, HDL-C exhibits both anti-inflammatory and antioxidant effects, which can reduce neuronal injury [66–68]. Finally, HDL-C preserves the integrity of the blood-brain barrier by suppressing platelet aggregation and leukocyte adhesion in the vascular endothelium [69, 70], which is beneficial for both neural and cognitive functions. Our finding of sex-specific association between HDL-C and the severity of psychotic symptoms adds valuable knowledge to the growing literatures on the overall relationship between serum lipids and psychotic symptoms [59, 71, 72]. This association is potentially multifactorial, with one possible factor being sex hormones, especially estrogen [73, 74]. The observed strong association between HDL-C and psychotic symptoms, though cross-sectional, supports investigating whether metabolic interventions can improve psychotic outcomes.

We demonstrated that MetS significantly correlated with the prevalence and severity of psychotic symptoms in BD males only. The concept of MetS was originally established to identify patients at an increased risk of cardiovascular diseases. Compared to the general population, patients with BD exhibit an increased risk of MetS [75, 76]. Previous studies have shown that there is a bidirectional relationship between the prevalence and severity of MetS, and psychotic symptoms [77]. Studies conducted in France [78] and China [79] show that the risk of MetS among men was approximately twice compared to that among women, affecting the sex-specific phenomenon of psychotic symptoms. This correlation is potentially associated with inflammation [80, 81], autoimmune response [82, 83], and unhealthy lifestyles [84, 85]. Moreover, the sex differences may be associated with dysfunction of the oxytocin (OT) system, thereby significantly contributing to the etiology of psychotic symptoms and MetS [86], Our findings suggest that for male patients with BD, routine monitoring of MetS indicators (e.g., waist circumference, blood lipids) may help identify individuals at higher risk for psychotic symptoms.

Our study found that the TSH levels was associated with the prevalence and severity of psychotic symptoms in BD females only. Prevalence of thyroid dysfunction was higher in patients with BD compared to healthy individuals, with hypothyroidism being the primary manifestation, and this trend is particularly pronounced among female patients [87]. Our results are consistent with previous research showing that higher TSH levels are associated with psychotic symptoms in emotional disorders [88–90]. Female BD patients are prone to thyroid dysfunction and induce psychotic symptoms, owing to abnormalities in the central endocrine system. Thyroid-brain crosstalk may mediate psychotic symptoms via the hypothalamus-pituitary-thyroid (HPT) axis [91], hypothalamic-pituitary-adrenal (HPA) axis [92], and secretion of thyroid hormone (TH), thereby affecting brain function, such as the neuroendocrine system [93], as well as the catecholaminergic and serotonin (5-HT) systems [94]. Besides, TSH was a bridge node connecting MetS and clinical symptoms, potentially affecting psychotic symptoms through the regulation of lipid metabolism [95]. In female patients, the association between thyroid function (TSH) and psychotic symptoms indicates that thyroid screening in women with mood symptoms may carry additional psychiatric significance.

Nevertheless, this study findings should be cautiously interpreted due to the following limitations: First, this study employed a cross-sectional design, which provides no insight into whether these sex differences evolve throughout the entire disease process. Therefore, in-depth longitudinal research is required to elucidate the causal relationships. Second, our results should be considered as preliminary findings owing to the absence of healthy controls. Third, this study did not account for potential confounding effects of female sex hormonal status and menstrual status, which may influence psychotic symptoms in BD [96]. Finally, the limitation concerns statistical power regarding the negative finding mentioned and these inconsistencies may be due to methodological heterogeneity—such as study population and diagnostic thresholds. Despite these limitations, this study exhibits various advantages, including employing a large sample of FEDN BD patients within the Han Chinese cohort, and identifying sex differences in psychotic symptoms in patients with BD, which may contribute to suggesting potential research directions. Therefore, we suggest that future longitudinal prospective studies adopt a more homogeneous approach to measure sex differences in more balanced groups, including a healthy control group, and critical factors such as mood state, menstrual cycle phase, and the reproductive aging status should be considered.

## Conclusions

This is the first study among the Han Chinese population with BD to demonstrate the following: (1) No significant sex difference was observed in the prevalence of psychotic symptoms; (2) HDL-C remains a significant protective factor against psychotic symptoms in both sexes, however, it is only correlated with the severity of psychotic symptoms in females; (3) The risk of psychotic symptoms is regulated by sex-specific indicators—including MetS which is predominant in males, with TSH characteristic in females. In conclusion, this study highlights sex-divergent pathways linking metabolic dysfunction (in males) and thyroid axis markers (in females) to psychotic symptoms in early-stage BD. These findings advocate for a sex-stratified approach in the clinical assessment of BD, suggesting that routine metabolic and thyroid screening may help identify patients at greater need of monitoring for psychotic features. Although the prevalence of psychotic symptoms shows no sex differences, our findings underscore the potential interaction between sex, biological factors and clinical symptoms. This interaction may inform the development of sex-specific clinical treatment approaches aimed at reducing the illness burden associated with BD.

## Data Availability

The datasets used and analyzed in this study are available from the corresponding author upon reasonable request.

## Declarations

### Declaration **of AI and AI-assisted technologies in the writing process**

The authors declare that this article does not use AI and AI-assisted technologies.
